# Incidence of Catheter-Related Infections in Hospitalized Cardiovascular Patients

**DOI:** 10.5812/cardiovascmed.9388

**Published:** 2013-05-20

**Authors:** Kambiz Mozaffari, Hooman Bakhshandeh, Hadi Khalaj, Hengameh Soudi

**Affiliations:** 1 Rajaie Cardiovascular, Medical and Research Center, Tehran University of Medical Sciences, Tehran, IR Iran; 2 Cardiovascular Intervention Research Center, Rajaie Cardiovascular, Medical and Research Center, Tehran University of Medical Sciences, Tehran, IR Iran

**Keywords:** Catheter-Related Infections, Cardiovascular Diseases, Bacteremia, Hospitalization, Iran

## Abstract

**Background:**

Catheter Related Blood stream Infections (CRBSI) are prevalent and a potentially fatal complication pertaining to cardiovascular implant devices. There have been no major studies on bacterial colonization of catheters in cardiovascular patients in Iran.

**Objectives:**

To evaluate the incidence of catheter colonization of bacteria in the largest Iranian cardiovascular center.

**Patients and Methods:**

March 2011 to 2012, Cauterization procedures performed on 60 patients hospitalized in Rajaie Cardiovascular Medical and Research Center, Tehran, Iran, with arterial or venous catheterization, inserted 48 hours or more, catheter evaluations done by culture methods. Blood cultures were also obtained simultaneously.

**Results:**

Forty-four out of 60 catheters (73.3%) were positive with a significant colony count. Of 44 positive cases, 11 patients had positive blood culture. Three most frequently isolated microorganisms were Staph Albus [14 (32%)], Entrococcu [12 (27%)] and Acinetobacter [5 (11%)]. gram-positive cocci were sensitive to Vancomycin and Linezolid and gram-negative bacilli were sensitive to Amikacin, Gentamicin, Tobramycin and Imipenem.

**Conclusions:**

The study findings revealed that the catheter infection in our patients had sources other than normal skin flora. These results will assist in determining the possible source of the infections, furthermore, how they are transmitted, moreover aid in controlling and preventing these dangerous in- infections.

## 1. Background

Vascular devices are extremely common in modern healthcare systems, particularly in Intensive Care Units (ICU’s). Such catheterizations are requisite to achieving acceptable vascular access; however, they pose some complications such as local site infection, and Catheter Related Blood stream Infections (CRBSI) and so forth (1). The incidence rate of serious complications with central venous catheters (CVC’s) is greater than that of peripheral catheters, particularly in those used for critically ill patients. These devices can become dangerous and fatal sources of infection if the recommended precautions are not considered by health-care providers (2). Catheter related bloodstream infections is a common, expensive and yet fatal consequence of catheterization. In the USA, the annual incidence of CRBSI is 80,000, leading to 28,000 deaths at a cost of 2.3 billion dollars (3). Several studies on intravascular colonization in surgical and ICU patients have been concluded. In one case control survey, 5% of ICU patients had catheter related bacteremia, with 1.5% presenting as CRBSI5. These infections begin either as colonization of the catheter by skin flora or as a local infection of the intradermal portion of the catheter site. Organisms may also enter via a contaminated hub or infusion set. Microorganisms adhere to the catheter surface and extend on to the part of the catheter within the vasculature. From Here, colonizing organisms may be swept away into the bloodstream and seed in the distant tissues of the body. Common pathogens involved include coagulase negative staphylococci (4), Staphylococcus aureus, yeasts, enterococci and various gram-negative bacilli. The number of patients on home intravenous therapy is on the increase, predominantly for total parenteral nutrition or cancer chemotherapy. At the same time, these devices are increasingly associated with sepsis and are now the commonest cause of bloodstream infections, causing significant morbidity and mortality (4). Because there has been no extensive study on bacterial colonization of arterial and venous catheters in cardiac surgery or intensive care unit patients in Iran it was decided to evaluate the incidences of catheter colonization by such bacteria in the Rajaie Medical Center.

## 2. Objectives

This study was a laboratory-based evaluation of the microbiological status of infections to provide epidemiological information about the most frequent pathogens in our cardiovascular tertiary health care center.

## 3. Patients and Methods

Sixty patients hospitalized in our center for arterial or venous catheterization, were evaluated in this study. The catheters, which were inserted for more than 48 hours, were assessed by culture methods. The study was performed in Rajaie Cardiovascular Medical and Research center, Tehran, Iran, from March 2011 to 2012. Catheter related blood stream infections were considered if there was bacteremia or fungemia in a patient who had an intravascular catheter with at least one positive blood culture obtained from a peripheral vein with clinical manifestations (i.e., fever, chills, and/or hypotension) and no apparent source of the bloodstream infection except the catheter. Patient samples were sent to lab for culture and other lab tests.

### 3.1. Steps in Specimen Collection

1. The catheter sites were first thoroughly cleansed in each patient.
2. With the aid of sterile forceps, each catheter was carefully removed, making sure it did not touch the skin.
3. With sterile scissors, at the terminal 4-5 cm of the catheter was cut into a sterile container.
4. The specimen was transported to the laboratory within one hour.

### 3.2. Steps in Culture of Samples

1. All the catheter tips were processed in the Thioglycolate broth for 24 hours.
2. Subculture on four media was performed including two blood agar (aerobic and anaerobic conditions respectively), MacConkey agar and chocolate agar (incubated in a candle jar).
3. A colony count of > 15 on each plate was considered significant and was further processed.
4. The inoculated plates were incubated aerobically at 37 o C for 24hrs except chocolate agar plates, which were incubated overnight in the candle jar at 37 o C. If plates showed no growth after 24 hours, they were incubated for another 24 hours.

### 3.3. Identification of Positive Cases

Smears were prepared from plates showing growth and were stained by Gram's Method. Gram positive cocci in clusters were tested by catalase and tube coagulase tests. Catalase and coagulase positive strains were identified as Staphylococcus aureus. Catalase positive and coagulase negative strains were Coagulase negative staphylococci (5) and further differential tests including Sucrose fermentation, Ornithin decarboxylation and urease were used. gram-negative bacilli were identified up to species level as in the standard protocol described by Mackie and McCartney.

### 3.4. Antibiotic Susceptibility Testing 

Antibiotic susceptibility testing of all the isolates was performed according to the Kirby-Bauer disc diffusion method, using Mueller-Hinton agar (MHA). The following antibiotic discs were used (provided by Hi-media, Padtan teb, ABTEK, ROSCO):

Penicillin (10U), Ciprofloxacin (5µg), Cephalexin (30µg), Amikacin (30µg), Gentamycin (10µg), Tobramycin (10µg), ceftriaxone (30µg), cefepime (30µg), Imipenem (10µg), Piperacillin (100µg), Ampicillin (10µg), Co-Amoxiclav (30µg), Vancomycin (30µg), Cefoxitin (30µg), and Linezolid (30µg), cefazolin (30µg), Chloramphenicol (30µg), Ofloxacin (5µg), Meropenem (10µg). Cefoxitin was used to determine the Methicilin-Resistant S.aureus (MRSA) strains.

## 4. Results

Sixty catheters were removed and sent to our laboratory as follows: fifty-six central vein, one arterial, one femoral vein, and lastly, two Shaldon catheters. Forty-four cases (73.3%) were positive with a significant colony count. In this group, the male/female ratio was 1 (22 cases in each group). Of 44 positive cases, 11 patients had positive blood culture. In 4 cases, the microorganisms isolated from blood culture were in accordance with the catheter culture results. Two cases of positive blood culture were candida species, and the third one was Acinetobacter species and the last one was Enterococci. It seems that an important relationship could be observed between the results of catheter culture and blood culture. According to our observations, culture positivity was similar in both genders. In this study, eleven patients were from ICU, nine from post-CCU, sixteen cases were from CCU, with only seven cases from general surgery wards. The average age of patients, regardless of the culture result was 42, whereas thirteen cases with positive results were under the age of five. There were 8 cases with mixed culture results (in whom multiple organisms were seen), including: enterococci, S. albus, candida sp., S. aureus, acinetobacter Spp. and proteus Spp. The most frequently isolated microorganisms in our patients are listed in Table 1. Antibiotic sensitivity tests showed gram-positive cocci to be sensitive to vancomycin and linezolid and gram-negative bacilli were sensitive to amikacin, gentamicin, tobramycin and imipenem.

That are resistant to many of the conventional therapeutic modalities.

Entrococci were sensitive to co- amoxiclav, and ampicillins. The details of antibiotic sensitivity of isolated organisms are given in Table 2. It is noteworthy that all cases positive for S.aureus were methicillin-resistant and most cases of entrococci were found to be vancomycin -resistant. Finally yet importantly, 5 cases (11%) of the catheters were positive for Acinetobacter sp, a leading cause of nosocomial infections.

**Table 1.** Organisms Isolated From Positive Cases

Isolated Organisms	No. (%)
*StaphAlbus*	14 (32)
*Entrococcu* *sp.*	12 (27)
*Acinetobacter* *sp.*	5 (11)
*Staphylococcus* *aureus*	4 (9)
*Candida sp.*	4 (9)
*Staphylococcus* *Coa. Negative*	4 (9)
*Candidaalbicans*	3 (6.8)
*P.aerogineasa*	2 (4)
*Escherichia coli*	1 (2)
*Klebsiella* *Sp.*	1 (2)
*Proteus sp.*	1 (2)
*Serratia* *sp.*	1 (2)

**Table 2.** Antibiotic Sensitivity Pattern of Isolated Organisms

	Isolated Organisms											
Antibiotic	*S.* *aureus*		*S.* *albus*		*Enterococci*		*Enterobacteriacae*		*Acinetobacter* *sp.*		*P.* *aeruginosa*	
	S^a^	R^a^	S	R	S	R	S	R	S	R	S	R
**Cefazolin**	-	4	12	2	-		-		-		-	
**Vancomycin**	4	-	14	-	1	11						
**Linozolid**	4	-	14	-	4	8						
**Chloramphenicol**	4	-	14	-	7	5						
**Amikacin**	13		11	3	-	12	2	-	-	5	1	1
**Cefoxitin**	-	4	14	-	-	-						
**Ampicillin**	-	-	-	-	7	5						
**Amoxicillin-Clavulanicacid**	-	-	-		7	5						
**Tobramycin**	-	4	10	4	1	11	2	-	5	-	1	1
**Ofloxacin**	-	-	-	-		2						
**Ceftriaxone**	-	4	5	9	-	12	1	1	-	5	1	1
**Ciprofloxacin**	-	4	9	5	1	11	3	1	-	5	1	1
**Meropenem**	2	2	8	6	7	5	1	1	-	5	1	1
**Penicillin**					1	11						
**Piperacillin**							1	1				
**Cephalexin**					-	12	-	4	-	5	12	
**Gentamycin**	-	4	9	5	1	11	3	1	-	5	1	1
**Cefepime**	-	4	9	5	-	12	2	2	-	5	-	2
**Imipenem**					1	11	4		1		11	

^a^ Abbreviations: R, Resistant; S, sensitive

## 5. Discussion

Nosocomially-acquired blood stream infections are one of the most serious and potentially life threatening conditions in patients undergoing cardiac surgery. Early diagnosis and therapy are the cornerstones in the prevention of morbidity and mortality (5). Prediction of pathogens responsible for growth and their antimicrobial resistance pattern are crucial for successful therapy. In the present study, the rate of catheter colonization was 73% (44 out of 60) which seems to be high. S.epidermidis or s.albus was found to be the most frequent isolate (32%) followed by Enterococcus (27%). *Acinetobacter* *sp*, *S.* *aureus*, candida sp, Candida albicans, P.aeruginosa K.pneumoniae, E coli, proteus sp and serratia sp were isolated in descending order of frequency. This finding was similar to the study by Mohan et al in which *S.* *epidermidis* was the most commonly isolated species (82.3%) (6). Whereas in the study done by Jain et al, S.aureus was shown to be the most frequent isolate followed by Candida, Coagulase-negative Staph, pseudomonas, *klebsiella* *sp* and E. coli (7). In catheter related infections skin associated microorganisms are the predominant pathogens. Coagulase negative staphylococci [*S.* *epidermidis*] are the commonest, possibly because they have the best adherence to inert surfaces. On the other hand, S.aureus infections are second in frequency, with the infection risk being highest in patients with neutrophil defects or thrombophlebitis (8), However this is in contrast to our finding which revealed Enterococci to be second in rank to S.albus. Enteric germs such as enterococci, pseudomonas, candida species and *E. coli*, may colonize catheters either by contaminating skin or by hematogenous seeding from mucosal breaches (9). Colonized on the skin, organisms enter during catheter insertion or as a result of contamination by health care providers’ hands (5). The microbiological diagnosis of catheter related infection is very important, because therapy varies based on the microorganism isolated and its resistance pattern. *S.* *epidermidis* and other coagulase-negative Staphylococci usually adhere to the surfaces of the catheters to form slime/glycocalyx after catheter insertion into the vascular system (10). Colonization of microorganisms at the catheter insertion site was an independent risk factor, showing the importance of skin as a reservoir for catheter tip colonization (9). In our study, all staphylococci were susceptible to vancomycin and linezolid, a finding similar to the studies by Pathak et al. and Goel et al. (11, 12). Methicillin resistance among *s.* *aureus* isolates was seen in all of our cases (MRSA). We also found that 91.7% of enterococci were resistant to vancomycin (VRE), and 66.7% were sensitive to linezolid. Finally, 41.6% were sensitive to Ampicillin. Arias et al. reported resistance rates among enterococci for ampicillin to be 9.7%, and all enterococci showed susceptibility to linezolid (13). In our study, bacterial colonization was the same in both sexes in this study. Nasia et al. observed that the rate of catheter infection was more common in females and it was considered as a risk factor for catheter colonization (13). Statistics show the CCUs to be among the most important wards in heart centers, hence the higher rate of positive cases. In this survey, the incidence of acinetobacter (11%) was relatively high in comparison with other studies. Acinetobacter is transmitted through person-to-person contact, contact with contaminated surfaces, and even following environmental exposure. Acinetobacter can last quite some time on the hands of healthcare providers and in the environment on counters and other areas within healthcare settings. Healthcare providers who are colonized with this organism can spread the infection while not exhibiting clinical manifestations of the infection. Our findings suggested that possible relationship between catheter colonization and simultaneous positive blood culture result should be considered. Other studies in this field show that simultaneous positive blood culture is variable in patients with positive catheter culture and differs from 8.7% to 33% (9-11, 13, 14). As our study is solely a laboratory-based evaluation of the microbiological status of infections, we did not intend to include the patients’ clinical symptoms and manifestations here. Another reason for this limitation, if that could be named one, was that we did not have free access to the patients’ medical records provided by clinicians, retrospectively. Catheter-related blood stream infections and sepsis are common complications of modern medical therapy. Reduction of these complications may be achieved by minimizing intravenous access. If there is no absolute need for intravenous access, they should be avoided. Strict aseptic precautions while inserting catheters and careful monitoring for signs of inflammation will greatly reduce the risk of this iatrogenic infection. Although advances in diagnosing, preventing, and managing intravascular catheter-related infections have been demonstrated, this frequent clinical problem could still benefit from future breakthroughs. Accurate diagnostic techniques, especially while the suspected indwelling catheter is still in situ, can identify CVCs that should be removed while sparing others. It seems that the reemergence of gram-positive cocci and acinetobacter sp. is the predominant cause of nosocomial infections. In addition, in each of the genera of gram-positive cocci and acinetobacter sp. causing these infections, notable antimicrobial resistance is present. It is difficult to distinguish whether the morbidity and mortality rates in patients that are associated with infection caused by antibiotic-resistant species (as compared with their antibiotic-susceptible equivalents) relate to features that are intrinsic to the resistant strains and available therapy or to characteristics that are unique to the patients infected with each strain. Nevertheless, it seems obvious that options for effective antimicrobial therapy are becoming increasingly limited. Furthermore, in time, the absence of effective therapy will cause morbidity and mortality to increase. Thus, there are mandates to develop new effective antimicrobial therapies for infections caused by gram-positive cocci and Acinetobacter sp. to intensify efforts to limit the spread of resistance in these organisms, and to reduce the nosocomial transmission of and infection with these organisms.
